# Essential Oil of Carvone Chemotype *Lippia alba* (Verbenaceae) Regulates Lipid Mobilization and Adipogenesis in Adipocytes

**DOI:** 10.3390/cimb44110389

**Published:** 2022-11-18

**Authors:** Katherin Bonilla-Carvajal, Elena E. Stashenko, Natalia Moreno-Castellanos

**Affiliations:** 1 Department of Basic Sciences, Health Faculty, Universidad Industrial de Santander, Bucaramanga 680002, Colombia; 2 Centro Nacional de Investigaciones para la Agroindustrialización de Especies Vegetales Aromáticas y Medicinales Tropicales/CENIVAM. Chemistry School, Science Faculty, Universidad Industrial de Santander, Bucaramanga 680002, Colombia; 3 Research Group-Centro de Investigación en Ciencia y Tecnología de Alimentos/CICTA, Department of Basic Sciences, Health Faculty, Universidad Industrial de Santander, Bucaramanga 680002, Colombia

**Keywords:** obesity, adipocyte, essential oil, *Lippia alba*, insulin resistance, carvone

## Abstract

Obesity is characterized by an expansion of adipose tissue due to excessive accumulation of triglycerides in adipocytes, causing hypertrophy and hyperplasia, followed by hypoxia, alterations in adipocyte functionality, and chronic inflammation. However, current treatments require changes in lifestyle that are difficult to achieve and some treatments do not generate sustained weight loss over time. Therefore, we evaluated the effect of the essential oil (EO) of *Lippia alba* (Verbenaceae) carvone chemotype on viability, lipid mobilization, and adipogenesis of adipocytes in two normal and pathological cellular models in vitro. In 3T3-L1 adipocytes, a normal and a pathological model of obesity were induced, and then the cells were treated with *L. alba* carvone chemotype EO to evaluate cell viability, lipid mobilization, and adipogenesis. *L. alba* carvone chemotype EO does not decrease adipocyte viability at concentrations of 0.1, 1, and 5 µg/mL; furthermore, there was evidence of changes in lipid mobilization and adipogenesis, leading to a reversal of adipocyte hypertrophy. These results could be due to effects produced by EO on lipogenic and lipolytic pathways, as well as modifications in the expression of adipogenesis genes. *L. alba* carvone chemotype EO could be considered as a possible treatment for obesity, using the adipocyte as a therapeutic target.

## 1. Introduction

Adipocytes are endocrine cells of adipose tissue (AT) that store energy in the form of triglycerides within lipid droplets and release adipokines that participate in homeostatic balance, which are crucial in vascularization and immune response. However, when there is excess energy, adipocytes increase in size (hypertrophy) and quantity (hyperplasia), and adipogenesis and lipid mobilization are altered, causing changes that compromise adipocyte functionality and lead to the development of obesity [1,2].

Specifically, alteration of adipocyte functionality such as lipid mobilization leads to alterations in triglyceride accumulation (lipogenesis) and its positive stimulation pathways such as lipoprotein lipase (*Lpl*), insulin, and leptin, as well as its inhibition pathways such as AMPK [3,4,5,6]. Additionally, triglyceride hydrolysis (lipolysis) and its activation pathways such as hormone-sensitive lipase (HSL), triglyceride lipase (ATGL), and monoglyceride lipase (MGL), and the inhibitory pathways such as lipase, insulin, and AMPK, are altered as well [4,5,6,7]. In this context, altered lipid mobilization favors the adipocyte disbalance which, together with hypertrophy and hyperplasia, induces reticulum and mitochondrial stress in adipocytes [8], together leading to a state of chronic inflammation that evokes altered adipokines secretion, fibrosis, hypoxia, and induction of cell death [1].

All these modifications lead to the development of obesity pathogenesis. It is considered an epidemic disease with great importance in public health due to the frequency of its presence in the world population and the economic cost it represents for the health systems of different countries [9]. In addition, obesity is currently considered an epidemic of the 21st century since it is expected to maintain a constant increase in its rates in children, adolescents, and adults [9]. 

Therapies currently used to reduce obesity are mainly based on lifestyle modifications, medications, and bariatric surgery. However, most of them do not induce a sustained weight reduction over time, generating high costs for different countries, individuals, and public health [8]. In this line, it is important to search for new methods that can reduce the effects on adipocyte morphology and functionality as a target to treat obesity. Therefore, natural pharmacological interventions for obesity are necessary and well recognized by the clinical community. In this sense, therapies aimed at reversing hypertrophy, hyperplasia, and reversing the adipocyte effects of obesity may potentially affect obesity management.

The essential oils (EOs) distilled from aromatic plants have been confirmed to provide an effect like current therapeutic approaches, and previous studies demonstrated the reversal of obesity in Wistar rats fed high carbohydrate diets [10]. EOs have many biological properties, including antioxidant activity from having redox properties, allowing them to neutralize free radicals, and anti-inflammatory, by inhibiting the release of histamine and the activation of inflammatory mediators [11,12,13,14]. Recently, the effects of some compounds present in medicinal plants on adipocytes have been evaluated with a favorable effect on these cells [15,16,17,18,19,20,21,22,23,24,25,26,27], adipocyte number, and oxidative stress [28,29,30,31]. Moreover, recent data indicate that aqueous extracts of some EOs could improve metabolic alterations [15,32]. 

Some of these plants have been shown to have antidiabetic, antioxidant, and anti-inflammatory properties [3,6,10,11,13,14,15,16,17,18,19,20,21,22,23,24,25,26,27,28,29,30,31,32,33], including *Lippia* spp, which has been confirmed in previous studies to influence adipocyte functionality by decreasing lipogenesis, reversing the hypertrophic state, and generating changes in lipolysis and adipogenesis [33]. Specifically, *Lippia citriodora* showed an effect on some important genes in adipogenesis (CCAAT/enhancer-binding protein Alpha (*Cebpa)* and Peroxisome Proliferator- activated receptor gamma (*Pparg*)) and, therefore, modifies lipid accumulation, decreases adipocyte hypertrophy, and affects adipokine release after treatment for 48 h at concentrations of 50 to 400 µg/mL in 3T3-L1 adipocytes [33]. In addition, *Lippia alba* is known in traditional medicine for its antidiarrheal, antifungal, anticholesterolemic, and analgesic properties [33]. Previous studies have also confirmed its anti-inflammatory/antioxidant properties [32], and hypocholesterolemia properties with high anti-obesity potential have been demonstrated [15]. Likewise, previous research has shown that *L. alba* chemotypes present many monoterpenes, which have been shown to affect the mevalonate pathway through which different molecules such as cholesterol are produced and, therefore, could have a hypolipidemic capacity [33]. Therefore, considering the properties of *L. alba* and the importance of adipocytes in the development of the pathophysiology of obesity, this study proposes for the first-time adipocytes as a target using *L. alba* carvone chemotype for obesity treatment.

## 2. Materials and Methods

### 2.1. Characterization

#### 2.1.1. Sample Preparation

*Lippia alba* (Verbenaceae family) plants were grown under controlled agricultural conditions (temperature 26–28 °C, relative humidity 75–80%) in the experimental plots at CENIVAM (*Centro de Investigación para agroindustrialización de especies vegetales aromáticas y medicinales tropicales*) in the Industrial University of Santander (Bucaramanga, Colombia). The vegetable material belonging to the *L. alba* carvone chemotype was gathered in the flowering phenological stage and only healthy, undamaged plants (stems, leaves, and flowers) were used for the hydro-distillation carried out for two hours in a classical Clevenger apparatus, as described elsewhere [19]. Essential oil (0.8% yield) was dried with sodium sulphate, weighed (50 mg), and dissolved in dichloromethane (1 mL) for chromatographic analysis. 

#### 2.1.2. GC/MS Analysis

Analysis was performed on a GC 6890 Plus gas chromatograph (Agilent Technologies, Palo Alto, CA, USA), equipped with a mass selective detector MSD 5973 Network (Agilent Technologies, Palo Alto, CA, USA) that used electron ionization [(ionization with electrons (IE), 70 eV]. Helium (99.995%, AP gas, Messer, Bogotá, Colombia) was used as carrier gas, with initial inlet pressure of 113.5 kPa; its volumetric flow rate was kept constant (1 mL/min) during the chromatographic run. The injection volume was 2 μL in split mode (30:1) and the injector temperature was kept at 250 °C.

Compound separation was carried out in two capillary columns, one with polar stationary phase of poly (ethylene glycol) (DB-WAX, J & W Scientific, Folsom, CA, USA) of 60 m × 0.25 mm (i.d.) × 0.25 μm (d_f_) [internal diameter, mm (i.d.), and stationary phase thickness, µm (df)] and another with non-polar stationary phase phenyl-poly(methyl siloxane), 5%-Ph-PDMS (DB-5MS, J & W Scientific, Folsom, CA, USA) of the same dimensions. The oven temperature was programmed from 50 °C (5 min) to 150 °C (7 min), at 4 °C/min, and then up to 230 °C (50 min) at 4 °C/min when the polar column was used. With the non-polar column (DB-5MS), the temperature of the chromatographic oven was programmed from 45 °C (5 min) to 150 °C (2 min) at 4 °C/min, then up to 300 °C (10 min), at 5 °C/min. The temperature of the GC-MS transfer line was set at 230 °C when the polar column was used, and at 300 °C for the non-polar column. The temperatures of the ionization chamber and the quadrupole were 250 °C and 150 °C, respectively. The mass range for the acquisition of ionic currents was mass/charge ratio (*m/z*) 45–450 µ, with an acquisition speed of 3.58 scan/s. Data were processed with MSDChemStation G1701DA software (Agilent Technologies, Palo Alto, CA, USA). Compound identification was based on their linear retention indices (LRI), calculated from the retention times of the compound of interest and those of the C_6_–C_25_ and C_8_–C_40_ *n*-alkanes present in commercial mixtures (Sigma-Aldrich, San Luis, MO, USA), according to Equation (1):

(1) $$\mathrm{LRI}=(100\;\times \;n)+100\;\times \left[\frac{t_{\mathrm{Rx}}-{\;t}_{\mathrm{Rn}}}{t_{\mathrm{RN}\;}-{\;t}_{\mathrm{Rn}}}\right]$$

LRI = Linear retention index for the compound of interest.
n, N = Number of carbon atoms of the *n*-alkane that elutes before (n), or after (N) the compound of interest (x).
t_RX_ = Retention time of the compound of interest (min).
t_RN_, t_Rn_ = Retention times of the *n*-alkanes that elute before (n) or after (N) the compound of interest (x) (min).

For tentative identification, experimental mass spectra were compared to those in Adams (2007), NIST (2017), and Wiley (2008) spectral databases. Confirmatory identification of some compounds was obtained by comparison of their LRIs and mass spectra with those of available standard substances.

### 2.2. Cell Culture

Mature 3T3-L1 adipocytes obtained from the American Type Culture Collection (ATCC CRL-3242) were seeded at 3 × 10^3^ cells/cm^2^ and cultured according to the protocol described previously [34]. Briefly, adipocyte medium containing Dulbecco’s Modified Eagle’s Medium [Dulbecco′s Modified Eagle′s Medium (DMEM, Sigma-Aldrich, San Luis, MO, USA)] with 100 mg/dL glucose (*v*/*v*), 2% L-glutamine (*m*/*v*), 1% antibiotic–antimycotic (*v*/*v*), 1.5 g/L sodium bicarbonate (*m*/*v*), and 10% fetal bovine serum (*v*/*v*) (FBS, Gibco, Thermo Fisher Scientific, Waltham, MA, USA) was added. All cultures were incubated at 37 °C, with 5% CO_2_, until 80% confluence; after 3 days of culture, differentiation was initiated using DMEM enriched with 0.5 mmol/L 3-isobutyl-1-methylxanthine [IBMX, (Sigma-Aldrich, San Luis, MO, USA)], 0.2 μmol/L (*m*/*v*) dexamethasone (Sigma-Aldrich, San Luis, MO, USA), 2 µmol/L (*m*/*v*) rosiglitazone, and 1 µg/mL (*m*/*v*) insulin. After the 3-day induction period, the medium was changed every 3 days with DMEM but without IBMX, rosiglitazone, and without dexamethasone during the remaining 8 days of adipocyte differentiation. On day 10 of differentiation, all experiments were performed. For all the experimental tests, the seeding density of 3000 cells/cm^2^ was used.

Mature adipocytes were treated or not with different concentrations (0.1, 1, 5, and 10 μg/mL) of EOs distilled from carvone chemotype *L. alba*. Simultaneously, the in vitro obesity model (high glucose and high insulin—HGHI) characterized and described before [35] was used to evaluate the same parameters as a pathological condition mimicking obesity in vitro with or without the EO.

### 2.3. High Glucose—High Insulin Induction Model (HGHI)

To induce the pathological model of obesity and insulin resistance in adipocytes in vitro, we used the protocol previously established in Bonilla-Carvajal et al., 2022 [35]. Briefly, DMEM medium was added with 450 mg/dL glucose (*v*/*v*) and insulin at 10^6^ pmol/L (*m*/*v*) for 24 h. 

### 2.4. Evaluation of Cytotoxicity by MTT

Mature adipocytes that were untreated (normal model) and those with a pathological state—HGHI (HGHI model)—were exposed to carvone chemotype *L. alba* EO at different concentrations of 0.1, 1, 5, and 10 µg/mL during 8 h; once the cells were treated with EO, the MTT [3-(4.5-dimethylthiazol-2-yl)-2.5-diphenyltetrazole bromide] assay was performed as previously described [34,36]. Briefly, the cells were treated with an MTT solution at a concentration of 0.5 g/L (*m*/*v*) for 5 h at 37 °C, then the MTT solution was removed and dimethyl sulfoxide (DMSO) was added. Finally, the absorbance was read at 570 nm in the spectrophotometer (Thermo Fisher Scientific, Waltham, MA, USA).

### 2.5. Cell Size

Briefly, following the protocol previously established by Bonilla-Carvajal et al., 2022 [35], pathological and normal model cells were exposed to carvone chemotype *L. alba* EO at different concentrations of 0.1, 1, and 5 µg/mL, and then the samples were stained with Oil Red O to measure the diameter by light microscopy. At least 30 adipocytes were selected by each experiment and per each replicate for cell size measurement. According to the protocol described above, cells were fixed in 4% (*m*/*v*) paraformaldehyde (PFA) at room temperature in phosphate-buffered saline [PBS 0.01 mmol/l, pH 7.4] for 15 min, washed with 60% (*v*/*v*) isopropanol (Sigma-Aldrich, San Luis, MO, USA) and stained with Oil Red O (Sigma-Aldrich, San Luis, MO, USA) for 30 min. The cells were observed with a Leica DM500 optical microscope (Leica, Wetzlar, Alemania) and the images obtained were analyzed with Image J software.

### 2.6. Lipogenesis (Lipid Synthesis) and Lipolysis (Lipid Breakdown) 

Triglyceride content (lipogenesis) and glycerol release (lipolysis) were measured in mature adipocytes treated or not with the HGHI model, following the previously described protocol [34]. Specifically, to evaluate lipogenesis, mature adipocytes previously exposed to the normal or pathological model and stimuli or not with carvone chemotype *L. alba* EO at different concentrations of 0.1, 1, and 5 µg/mL were used to measure lipolysis, and the cell culture medium was used to measure lipolysis. Once treated with the EO, the culture medium was removed and stored for lipolysis quantification. To evaluate lipogenesis, the adipocytes were washed with PBS 1X, then fixed with PFA at 4% (*m*/*v*), and once removed, with isopropanol at 60% (*v*/*v*), and were allowed to dry for 15 min. Finally, Oil Red O (Sigma-Aldrich, San Luis, MO, USA) (*v*/*v*) was added for 30 min, to be revealed with isopropanol at 100% (*v*/*v*). Finally, the samples were read at 540 nm (Thermo Fisher, Waltham, MA, USA).

Regarding lipolysis, in all the samples extracellular glycerol was measured using a colorimetric technique where a Free Glycerol Reagent (Sigma-Aldrich, San Luis, MO, USA) (*v*/*v*) kit (Sigma-Aldrich, San Luis, MO, USA) was used, as previously employed [22]. Finally, the glycerol reagent was added, incubated for 15 min at room temperature, and finally read at 540 nm (Thermo Fisher, Waltham, MA, USA). 

### 2.7. Adipogenesis

Mature adipocytes were exposed to both cellular models (normal and pathological) and subsequently treated with the carvone chemotype *L. alba* EO at different concentrations of 0.1, 1, and 5 µg/mL, and were used to perform adipogenesis evaluation. For this purpose, RNA extraction from adipocytes was performed using Trizol (Ambion-Invitrogen, Waltham, MA, USA) and purified with the RQ1 RNase-Free DNase kit (Promega, Madison, WI, USA). Integrity and quantification were performed using NanoDrop One (Thermo Fisher Scientific, Waltham, MA, USA), and gene expression of factors important in adipocyte phenotype was determined by real-time PCR. Primers and probes were designed using Biosearch Technologies software, using Forward (F) and Reverse (R) for preadipocyte factor 1 (*Pref-1*) F: TGCGTGGACCTGGAGAAAG, and R: TGGCAGGGAGAACCATTGATC, for Lipoprotein-lipase (*Lpl*) F: GGCTCTCTGCCTGAGTTGTAGAAAG and R: TCTTGGCTCTCTCTGACCTTGTTGA, for Peroxisome Proliferator- activated receptor gamma (*Pparg*) F: GAACCCAGAGTCTGCTGCTGATCTCTG and R: TCAGCGGGAAGGACTTTATGTATATG, and, finally, for CCAAT/enhancer-binding protein Alpha (*Cebpa*) F: GCGGGAACGCAACGCAACAACATC and R: CGGTCATTGTCACTGGTCAAC. Measurements were performed on days 0, 3, 6, and 10 of adipocyte differentiation using both cell models and treated or not with the EO. Cells were harvested for real-time PCR (BIORAD, CFX 96, Hercules, CA, USA) gene expression studies of the different markers of importance during adipocyte differentiation (*Lpl*, *Pref-1*, *Cebpa*, *Pparg*). All results were normalized for the expression of 18S rRNA (Thermo Fisher Scientific, Waltham, MA, USA). Quantitative data analysis was performed with Gen5 software (Agilent, Santa Clara, CA, USA).

### 2.8. Statistical Analysis

Data are expressed as the mean  ±  SEM of six replicates of at least three independent experiments. Statistical differences between mean values were analyzed using one-way ANOVA followed by Tukey’s post hoc test. A *p* value  <  0.05 was considered statistically significant. The statistical analyses were performed using the SPSS/Windows software (v15.0, Chicago, IL, USA) and GraphPad prism software (v7.0, La Jolla, CA, USA). 

## 3. Results

### 3.1. Characterization of the Essential Oil of Carvone Chemotype L. alba EO

The typical chromatograms of the *L. alba* carvone chemotype essential oil, obtained by GC/MS on both non-polar DB-5MS (60 m) and polar DB-WAX (60 m) columns, appear in Figures S1 and S2 of the Supplementary Materials. The relative GC (%) amount of each *L. alba* EO component is registered in Table 1, from which one can observe that the main EO compound was carvone (37.3%), followed by limonene (31.5%), germacrene (11.8%), and β-bourbonene (2.6%), as described elsewhere [19].

### 3.2. Viability

**Figure 1:**
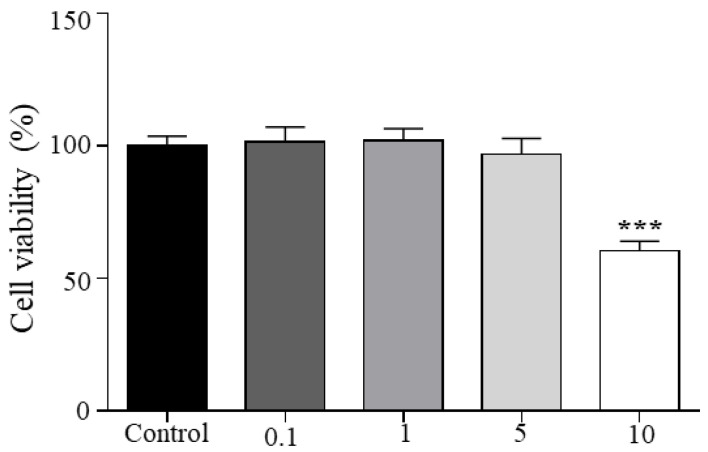
Cell viability of adipocytes treated with ascending concentrations (0.1, 1, 5, and 10 µg/mL) of *L. alba* carvone chemotype essential oil (EO) measured by MTT. Values are mean ± SEM. *** *p* < 0.001 vs. control (not treated with EO).

In our study we found, as evidenced in Figure 1, that cells treated with the EO studied at concentrations of 0.1, 1, and 5 µg/mL have cell viability higher than 96% compared to the control (not treated with EO). However, the concentration of 10 µg/mL produced in adipocytes a significant cell viability reduction (*p* = 0.0001), decreasing up to 40% compared to the control. Therefore, according to ISO 10993-5 [40], a moderate decrease in viability was obtained in adipocytes treated with a concentration of 10 µg/mL. However, with the concentrations of 0.1, 1, and 5 µg/mL, we did not identify a cytotoxic effect on adipocytes.

### 3.3. Cell Size 

**Figure 2:**
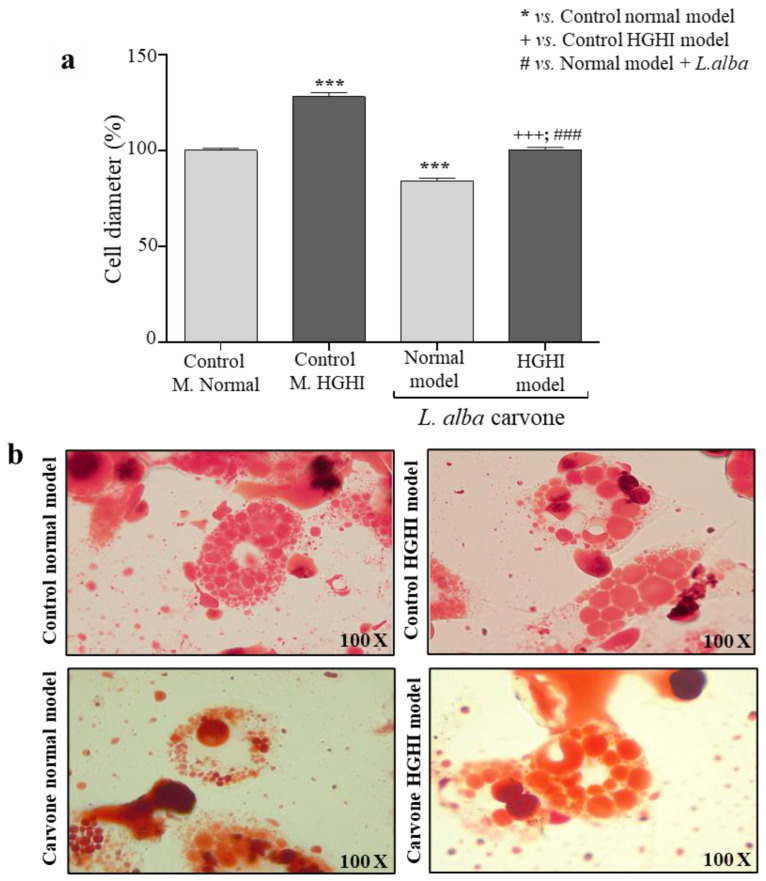
Adipocyte cell size. (**a**) Cell area percentage of adipocytes treated with 5 µg/mL of the carvone chemotype *Lippia alba* EO from normal and pathological model-HGHI; (**b**) triglyceride staining by Oil Red-O in normal adipocytes and the pathological model of obesity and insulin resistance (HGHI) in vitro, treated and untreated (control) with the carvone chemotype *Lippia alba* EO. *** *p* < 0.001 with respect to control model without EO; +++ *p* < 0.001 with respect to the pathological model without EO treatment; ### *p* < 0.001 with respect to control model treated with EO.

To identify changes after the EO treatment, cell size was measured (Figure 2a). Normal adipocytes treated with the EO showed a diameter of 0.12 mm (84%), while the control of the normal model without EO treatment had a diameter of 0.15 mm (100%). Therefore, the cell diameter in adipocytes treated with the carvone chemotype *L. alba* EO significantly decreased (*p* = <0.0001) up to 16% with respect to the control. Likewise, pathological adipocytes (stimulus with the HGHI model) showed a diameter of 0.14 mm (99%) after treatment with the EO, while the control for pathological adipocytes (not treated with the EO) obtained a diameter of 0.19 mm (128%). Therefore, the cell diameter decreased significantly (*p* = <0.0001) in the pathological adipocytes (HGHI model) by 28% after carvone treatment compared to the control of the HGHI model. 

Furthermore, this significant decrease in adipocyte diameter was also evident in Oil Red O-stained images (Figure 2b), where changes in the size and number of lipid droplets can be observed in both cell models after the *L. alba* EO treatment. Therefore, it was evident that treatment with carvone chemotype *L. alba* chemotype EO decreases adipocyte diameter in the normal model and the HGHI model. However, in the HGHI model, a greater reversal of adipocyte hypertrophy was evident.

### 3.4. Lipogenesis 

**Figure 3:**
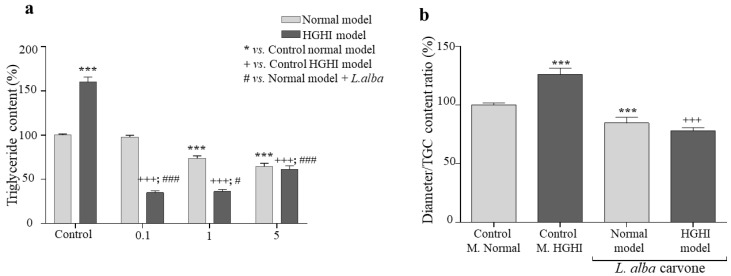
Lipogenesis of adipocytes treated with the carvone chemotype *L. alba* EO. (**a**) Intracellular accumulation of triglycerides after treatment with increasing concentrations (0.1; 1; 5 µg/mL) of the carvone chemotype *L. alba* EO in normal adipocytes and the pathological model of obesity and insulin resistance (HGHI) in vitro. (**b**) The ratio of cell diameter/TG content of adipocytes treated with 5 µg/mL of the carvone chemotype *L. alba* EO in normal and pathological models in vitro. *** *p* < 0.001 with respect to control model without EO; +++ *p* < 0.001 with respect to the pathological model without EO treatment; # *p* < 0.05; and ### *p* < 0.001 with respect to control model treated with EO.

We evaluated lipogenesis for which triglyceride accumulation in adipocyte lipid droplets was quantified. As shown in Figure 3, adipocyte lipogenesis significantly decreased after treatment with the carvone chemotype *L. alba* EO at concentrations of 1 (*p* = <0.0001; 73%) and 5 (*p* = <0.0001; 64%) µg/mL with respect to the control. Similarly, in pathological adipocytes (HGHI model), a significant decrease in lipogenesis was evidenced after treatment with the EOs at concentrations 0.1 (*p* = <0.0001; 35%), 1 (*p* = <0.0001; 36%), and 5 (*p* = <0.0001; 61%) µg/mL, with respect to the control. 

Furthermore, to confirm this decrease in triglyceride accumulation, the relationship between cell diameter and triglyceride accumulation was calculated (Figure 3b). We found a significant decrease after treatment with the carvone chemotype *L. alba* EO in normal adipocytes (*p* = 0.0007; 84%) and in pathological adipocytes (HGHI model) (*p* = 0.0006; 78%) with respect to the controls of each cell model. 

The above results suggest that treatment with the carvone chemotype *L. alba* EO produces a decrease in triglyceride accumulation in the adipocytes of the normal model and the pathological model of hypertrophic adipocytes (HGHI).

### 3.5. Lipolysis

**Figure 4:**
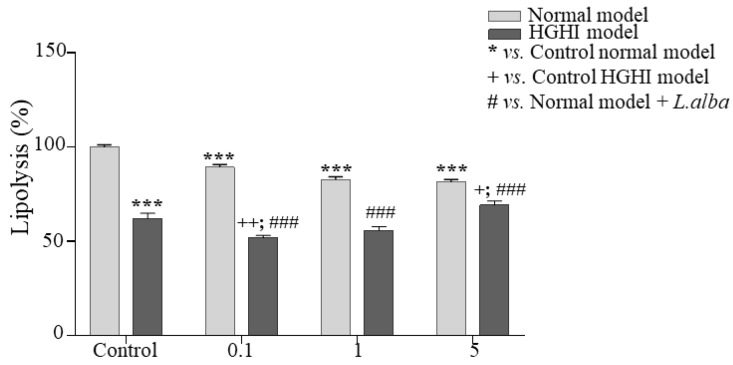
Lipolysis of the adipocyte. Free glycerol of the carvone chemotype *L. alba* EO. *** *p* < 0.001 vs. control (not treated with EO) of the HGHI model; *** *p* < 0.001 with respect to control model without EO; + *p* < 0.05; and ++ *p* < 0.01 with respect to the pathological model without EO treatment; ### *p* < 0.001 with respect to control model treated with EO.

To evaluate the lipid breakdown process, lipolysis was determined. We found that after treatment with the carvone chemotype *L. alba* EO, there was a significant decrease in lipolysis in normal adipocytes. Specifically, a dose-dependent lipolysis value of 89% at 0.1 µg/mL concentration (*p* = 0.0009), 82% at 1 µg/mL concentration (*p* = <0.0001), and 81% at 5 µg/mL concentration (*p* = <0.0001) was obtained, as shown in Figure 4. Furthermore, in pathological adipocytes (HGHI model), a reduction of lipolysis was also evidenced, obtaining a value of 51% at a concentration of 0.1 (*p* = 0.0013), compared to control cells (Figure 4). Therefore, the carvone chemotype *L. alba* EO generates a dose-dependent decrease in lipolysis both in normal and pathological adipocytes.

### 3.6. Adipogenesis

**Figure 5:**
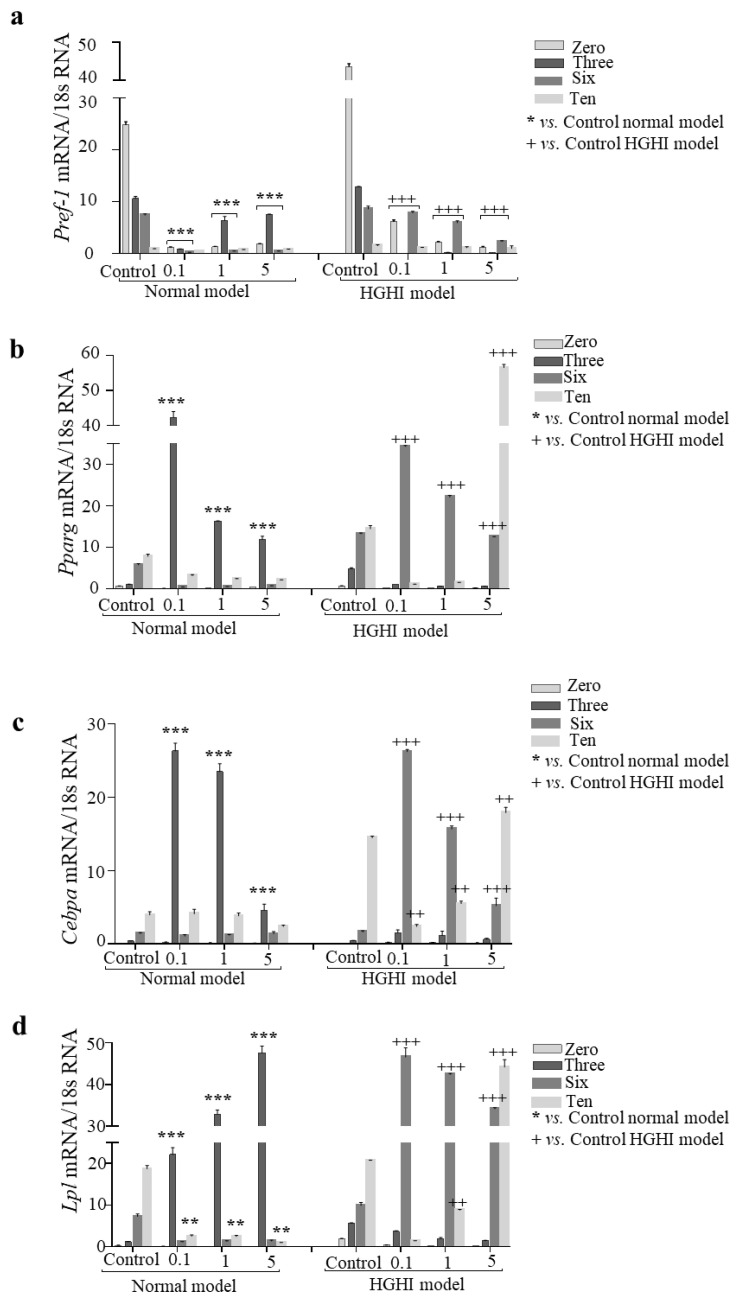
Adipogenesis. Expression of (**a**) *Pref-1*, (**b**) *Pparg*, (**c**) *Cebpa*, and (**d**) *Lpl* in adipocytes in normal and pathological models at differentiation days zero, 3, 6, 10 and treated or not with the carvone chemotype *L. alba* EO. ** *p* < 0.01; and *** *p* < 0.001; ++ *p* < 0.01; and +++ *p* < 0.001 with respect to the pathological model without EO treatment.

When evaluating the differentiation of adipocytes after treatment with the carvone chemotype *L. alba* EO, as shown in Figure 5a, for the adipocytes of the normal model, the expression of *Pref-1* decreased in all the concentrations of EO used (0.1, 1, and 5 µg/mL) in the different days of differentiation (0, 3, 6, and 10; *p = <*0.0001), compared to the control. Furthermore, in adipocytes previously treated with the HGHI model, there was also a decrease in *Pref-1* expression on all differentiation days evaluated after treatment with the carvone chemotype *L. alba* EO chemotype at concentrations 0.1, 1, and 5 µg/mL (*p = <*0.0001 for all the concentrations). Thus, the carvone chemotype *L. alba* EO negatively affects dose-dependent *Pref-1* expression in the normal model and the pathological model of obesity and insulin resistance.

Upon further evaluation of adipogenesis, we quantified *Pparg* expression (Figure 5b), finding that adipocytes in the normal model showed an increase in *Pparg* expression on day 3 of differentiation after treatment with the carvone chemotype *L. alba* EO in all concentrations used (0.1, 1, and 5 µg/mL; *p = <*0.0001). Likewise, in adipocytes previously treated with the HGHI pathological model, an increase in *Pparg* expression was evidenced at day 6 of differentiation in cells treated with the carvone chemotype *L. alba* EO at all concentrations used (0.1, 1, and 5 µg/mL; *p = <*0.0001) with respect to the control model. Therefore, the carvone chemotype *L. alba* EO increases *Pparg* expression at different days of adipogenesis in both cell models used. 

The expression of *Cebpa* was measured and we found, as shown in Figure 5c, that in the adipocytes of the normal model, the highest *Cebpa* expression was evidenced on day 3 in cells treated with EO at all concentrations used (0.1, 1, and 5 µg/mL; *p* = <0.0001). In addition, in the adipocytes of the pathological HGHI model, on day 6 an increase in *Cebpa* expression was observed after treatment with the carvone chemotype *L. alba* EO at 0.1, 1, and 5 µg/mL (*p* = 0.0001 for all the concentrations). Therefore, the carvone chemotype *L. alba* EO increases *Cebpa* expression in the normal model and the pathological model of obesity and insulin resistance. 

Finally, we determined the effect of the carvone chemotype *L. alba* EO on *Lpl* expression, finding, as shown in Figure 5d, that adipocytes from the normal model show an increase in *Lpl* expression on differentiation day 3 after treatment with the carvone chemotype *L. alba* EO at 0.1, 1, and 5 µg/mL treatment (*p = <*0.0001 for all the concentrations). Similarly, in the adipocytes of the pathological HGHI model, an increase in *Lpl* expression was evidenced on day 6 after EO treatment in all concentrations used (0.1, 1, and 5 µg/mL; *p = <*0.0001). Thus, EO increases *Lpl* expression in adipocytes of the normal model and the pathological model of obesity and insulin resistance. 

## 4. Discussion

Obesity is characterized by an expansion of adipose tissue due to hypertrophy and hyperplasia produced in adipocytes [1,2], causing alterations in adipocyte functionality and inflammation, which is directly related to metabolic disease [3]. Because of this, several studies focus their attention on natural treatments that have effects on the pathophysiology of the adipocyte and promote sustained weight loss over time [41]. Therefore, our study investigated the effects of the carvone chemotype *L. alba* EO on cell viability, lipid mobilization, and adipogenesis in normal adipocytes and in pathological adipocytes simulating a state of obesity and insulin resistance (in vitro HGHI model).

Previous studies have shown that carvone not only demonstrates antioxidant, antimicrobial, anticancer, and anti-inflammatory properties [20,21,22,42], but that the S-carvone isomer reduces obesity induced by a high-fat diet and insulin resistance; it inhibits the inflammation that causes obesity by blocking the expression of genes such as *Pparg*, transcriptional factor sterol regulatory element-binding protein 1 c (*Srebp1c*), acetyl-CoA carboxylase 1 (*Acc1*), and fatty acid synthase (*Fas*), which are important in the process of lipogenesis. [20].

Another important chemical component identified in the characterization of the carvone chemotype *L. alba* EO was limonene. In previous research, limonene has been shown to reverse hypertrophy in white and brown adipocytes, as well as to reduce the levels of triglycerides, total cholesterol, low-density lipoproteins (LDL), and glucose in the blood of mice [23]. It has also been shown that limonene improves mitochondrial biogenesis and lipolysis, and induces a phenotype like brown adipocytes, thus having effects on lipid metabolism [21,22,23,24]. Therefore, it could be possible that the chemical components of the carvone chemotype *L. alba* EO could be involved in the results obtained in this study by having effects on lipid mobilization and adipogenesis of adipocytes.

In our study, we found that a concentration of 10 μg/mL of EO significantly decreased viability. These results contrast with previous studies where *L. alba* was used and showing that higher concentrations (139.5, 164.9, and 375.5 μg/mL) maintain cell viability [43,44]. This could be explained by the cell lines used (HeLa and Vero cells) [44], which differ from the adipocytes used in this study. However, it is essential to note that lower doses of one of the carvone chemotypes (0.1, 1, and 5 μg/mL) do not affect adipocyte cell viability, this could be due to the major chemical components of the carvone chemotype *L. alba* EO, since previous studies using limonene (31.5%) [21] alone as a treatment in 3T3-L1 adipocytes did not affect cell viability [22,23].

Furthermore, recognizing that obesity produces a state of adipocyte hypertrophy, we evaluated the effects after treatment with the carvone chemotype *L. alba* EO, finding a decrease in adipocyte diameter and lipid droplet content, leading to the reversal of adipocyte hypertrophy. These changes in cell size could be because the amount of triacylglycerol (TAG) stored in the adipocyte lipid droplet depends on lipid mobilization, the balance between lipogenesis and lipolysis [4,5]. Therefore, it is evident that treatment with the carvone chemotype *L. alba* EO decreases adipocyte hypertrophy in both adipocyte models (normal and HGHI). It is notable that in the pathological model a greater reversal of adipocyte hypertrophy was evident. These results could be due to the chemical compounds of the carvone chemotype *L. alba* EO (37.3%) [21] because, in previous studies where monoterpenes such as limonene were used as treatment, inhibition of lipid accumulation and therefore a decrease in the size of adipocytes and lipid droplets was observed, by enhancing mitochondrial biogenesis, increasing HSL protein levels and causing changes in adipogenesis [4,21,24]. 

It is also important to note that when the intake of hypercaloric foods is exceeded, and other factors such as lack of physical activity are added, an excessive increase in lipogenesis is generated, leading to an imbalance of homeostasis and, in consequence, increasing adiposity, typical of obesity [4]. In our study, we observed that lipogenesis (lipid accumulation) of adipocytes of the normal and pathological HGHI model was significantly reduced; our results were like results previously described where the effect of different natural extracts such as *Fraxinus rhynchophylla* and *Oroxylum indicum* was evaluated, and where inhibition of lipogenesis and adipogenesis were observed [25]. This could be because *L. alba* EO causes chronic activation of adenosine monophosphate-activated kinase (AMPK) [26], which would decrease lipogenesis and promote adequate lipid mobilization [4]. This has already been demonstrated in previous studies where it was shown that limonene, an important chemical component of the carvone chemotype *L. alba* EO [21], has been shown to reduce the accumulation of triglycerides in adipocytes by increasing the expression of HSL (hormone-sensitive lipase), FAS, and AMPK [33]. Other plant extracts, such as *Citrus paradisi*, which has limonene among its active components, have been shown to regulate lipid metabolism through the expression of carnitine palmitoyl-transferase 1a (CPT1a) in an animal model [27]. Therefore, the above results suggest that the carvone chemotype *L. alba* EO induced a large decrease in triglyceride accumulation in both adipocyte models. However, it is relevant to note that in the lipogenesis study, a more significant decrease in triglyceride accumulation was observed in the pathological adipocytes after EO treatment. This could be explained by the fact that the carvone chemotype *L. alba* EO had a more substantial reduction of severe hypertrophy, as previously investigated with other extracts using in vivo and in vitro obesity models [28,29].

In this work, we were also able to determine the effect of the carvone chemotype *L. alba* EO on lipolysis, where we observed a decrease in lipolysis in both models. These results could be because treatment with the EO would influence the AMPK pathway, generating a decrease in lipogenesis and lipolysis [5,6] to maintain energy homeostasis, which is necessary for adipocyte survival. Briefly, AMPK activation would suppress lipolysis by inhibiting HSL, thus preventing diacylglycerol (DAG) hydrolysis [7]. Therefore, the carvone chemotype *L. alba* EO generates a dose-dependent decrease in lipolysis in adipocyte cell models. In addition, it is important to highlight that this decrease in lipolysis obtained in our study contrasts with other studies [21], where an increase in lipolysis in adipocytes after treatment with limonene was evidenced. However, this could be because limonene was only one of several chemical components that make up the carvone chemotype *L. alba* EO of this study, and therefore this effect would be caused by the synergistic behavior of all the components.

On the other hand, considering that adipogenesis is related to lipid mobilization in the adipocyte [41,45], we evaluated whether carvone treatment with the carvone chemotype *L. alba* EO influences adipogenesis in adipocytes from normal and pathological adipocytes in vitro. We evaluated whether carvone treatment with the carvone chemotype *L. alba* EO has an effect on adipogenesis in adipocytes from normal and pathological adipocytes in vitro, for which we quantified the expression of *Pref 1*, *Pparg*, *Cebpa*, and *Lpl* in normal and HGHI cell models.

When evaluating *Pref-1* expression, we found that after treatment with the carvone chemotype *L. alba* EO, there is a decrease in *Pref-1* expression in both models. This indicates that EO treatment could stimulate early adipocyte differentiation, both in the normal and the pathological state, as previous studies have shown that *Pref-1* was a marker of the pre-adipocyte state [46]. To continue with the evaluation of adipogenesis in the adipocyte, we found an increase in *Pparg* expression since day 3, and in the pathological model since day 6, with respect to the control. Considering that *Pparg* expression activity positively feeds back on *Pparg* activity, and that these two factors enhance each other’s expression and maintain the differentiated state [47,48], *Cebpa* expression was evaluated and had a similar behavior to *Pparg*. Previous studies have shown that not only is *Pparg* an activator of differentiation, but it also stimulates the expression of *Cebpa* [48] by performing a synergistic process in which, when expressed together, they stimulated adipogenesis [47,48]. Furthermore, this increased expression of *Pparg* induced adipocyte differentiation, while *Cebpa* participated in adipocyte proliferation and insulin sensitivity [49]. We concluded in our study that in pathological adipocytes there was a late differentiation of adipocytes compared to adipocytes of the normal model by increasing *Pparg* expression, which would stimulate *Cebpa* expression, which as previously demonstrated would lead to a higher activation of *Pparg* expression [47,49]. 

The above results were related to those observed when evaluating *Lpl* expression, where we found an increased expression of *Lpl* in the normal model since day 3 and in the pathological model since day 6, compared to the control. Taking into account that *Lpl* expression stimulates insulin sensitivity [50], this process of late differentiation of adipocytes in the pathological adipocytes is reflected in a decrease in lipogenesis and lipolysis; furthermore, in this process there could be involved the mitogen-activated protein kinase (MAPK)/extracellular signal-regulated kinase (ERK) signalling pathway of adipogenesis, which promotes the activity of *Cebpa* and *Pparg* activators, in addition to the AMPK pathway that has been shown to reduce *Pparg* and *Cebpa* expression. 

Therefore, treatment with the carvone chemotype *L. alba* EO influences adipocyte differentiation by inducing an increase in *Pparg*, *Cebpa*, and *Lpl* expression at different days of differentiation. This could be related to limonene in the carvone chemotype *L. alba* EO. From the results obtained in previous studies, it was evident that this chemical compound could positively regulate *Pparg* [23] and *Cebpa*, triggering an increase in adipogenesis through the Akt signalling pathway in adipocytes [22], which would lead to the reversal of the dysfunctional state of adipose tissue, and thus prevent the state of cellular senescence and inflammation characteristic of obesity. This is in addition to normalizing lipid mobilization, thereby regulating the production of adipokines and cytokines, and thus preventing the onset of metabolic disease, insulin resistance, and diabetes [22].

For all the above-described reasons, the results obtained in our study make it evident that treatment with the carvone chemotype *L. alba* EO substantially favors a reversal of the pathological state of obesity by influencing lipid mobilization, reduced hypertrophy, and, change the differentiation state of adipocytes. 

## 5. Conclusions

The carvone chemotype *L. alba* EO induces a reversion of the pathological state of the adipocyte, causing changes in lipid mobilization and adipogenesis. Therefore, the carvone chemotype *L. alba* EO could be considered as a possible treatment for obesity, using the adipocyte as a therapeutic target. These studies allow continuity to future in vivo studies, where the *L. alba* EO treatment could be evaluated in animal models of obesity, focusing on adipocytes.

## Figures and Tables

**Table 1:** GC/MS characterization of the essential oil obtained by steam distillation of aerial parts of *Lippia alba*, carvone chemotype (Verbenaceae family).

No.	Compound	Linear Rentention Indices				GC Relative Area, % DB-5MS	Identifi-Cation Criteria
		DB-5MS		DB-WAX			
		Exp.*	Lit.**	Exp.*	Lit.**		
1	*cis*-Hex-3-en-1-ol	854	850 [37]	1386	1380 [38]	0.3	a, b
2	α-Pinene	934	932 [37]	1022	1025 [38]	0.1	a, b, c
3	Camphene	952	954 [37]	1066	1069 [38]	0.2	a, b, c
4	Oct-1-en-3-ol	979	980 [38]	1451	1444 [38]	0.3	a, b, c
5	β-Myrcene	989	990 [37]	1163	1161 [38]	0.7	a, b, c
6	Limonene	1037	1029 [37]	1206	1198 [38]	31.5	a, b, c
7	*trans*-β-Ocimene	1047	1050 [37]	1252	1250 [38]	0.6	a, b
8	Linalool	1100	1096 [37]	1548	1543 [38]	1.0	a, b, c
9	*trans*-*p*-Mentha-2,8-dien-1-ol	1125	1122 [37]	1632	1639 [38]	0.3	a, b
10	*cis*-Limonene oxide	1140	1136 [37]	1465	1451 [38]	0.3	a, b, c
11	Borneol	1178	1169 [37]	1708	1700 [38]	0.8	a, b
12	*trans*-Dihydrocarvone	1209	1200 [37]	-	-	0.1	a, b
13	*cis*-Carveol	1229	1229 [37]	1838	1854 [38]	0.4	a, b
14	Carvone	1259	1258 [39]	1747	1734 [38]	37.3	a, b, c
15	Piperitone oxide	1262	1256 [39]	1725	1711 [39]	0.5	a, b
16	Piperitone	1264	1264 [39]	1737	1730 [38]	1.8	a, b
17	Geranial	1269	1270 [38]	1747	1725 [38]	0.4	a, b, c
18	Carvone oxide	1281	1273 [37]	1840	-	0.1	a, b
19	Thymol	1290	1290 [37]	2180	2164 [38]	0.5	a, b, c
20	*trans*-Carvyl acetate	1331	1342 [37]	-	1727 [38]	0.1	a, b
21	Piperitenone	1343	1343 [37]	1929	1909 [38]	1.7	a, b
22	α-Copaene	1382	1376 [37]	1501	1491 [38]	0.1	a, b
23	β-Bourbonene	1392	1376 [37]	1530	1523 [38]	2.6	a, b
24	β-Elemene	1394	1390 [37]	1598	1591 [38]	1.6	a, b
25	β-Ylangene	1427	1421 [39]	1585	1589 [39]	0.5	a, b
26	*trans*-β-Caryophyllene	1430	1427 [39]	1609	1599 [38]	0.4	a, b, c
27	β-Copaene	1439	1432 [39]	1585	1580 [38]	0.5	a, b
28	*trans*-β-Farnesene	1454	1456 [37]	1669	1664 [38]	1.4	a, b
29	Alloaromadendrene	1471	1460 [37]	1658	1650 [39]	0.7	a, b
30	Germacrene D	1493	1481 [38]	1725	1708 [38]	11.8	a, b, c
31	Bicyclogermacrene	1505	1500 [37]	1748	1735 [38]	0.7	a, b
32	δ-Cadinene	1524	1523 [37]	1762	1756 [38]	0.4	a, b
33	*trans*-α-Bisabolene	1544	1544 [39]	1775	1777 [39]	0.2	a, b

Identification criteria: a Tentative identification based on mass spectra (MS, EI, 70 eV, match > 90%); b Tentative identification based on LRI, determined on DB-WAX and DB-5MS columns ([37] Adams, 2007; [38] Babushok et al., 2011; [39] NIST 2017); c Confirmatory identification based on mass spectra and LRI, using standard substances. * Exp: Experimental. ** Lit: Literature.

## Supplementary Materials

The following supporting information can be downloaded at: https://www.mdpi.com/article/10.3390/cimb44110389/s1, Figure S1: Chromatographic profile (GC/MS, full scan) of *Lippia alba* carvone chemotype essential oil. DB-5MS (60 m) column, split 1:30, MSD (EI, 70 eV); Figure S2: Chromatographic profile (GC/MS, full scan) of *Lippia alba* carvone chemotype, essential oil. DB-WAX (60 m) column, split 1:30, MSD (EI, 70 eV).
