# Supplementary material for: Essential Oil of Carvone Chemotype Lippia alba (Verbenaceae) Regulates Lipid Mobilization and Adipogenesis in Adipocytes
Source: Curr Issues Mol Biol. 2022 Nov 18;44(11):5741–55. doi: 10.3390/cimb44110389 (PMC9688983; doi:10.3390/cimb44110389)
Supplement: Supplementary file 1 [file cimb-44-00389-s001.zip › cimb-1979774-supplementary.pdf]

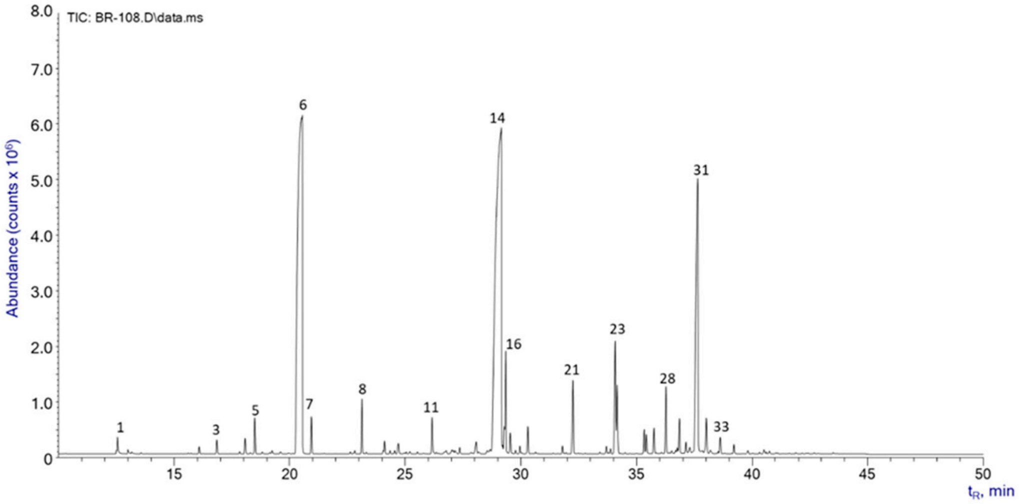

Figure S1. Chromatographic profile (GC/MS, full scan) of *Lippia alba* carvone chemotype essential oil. DB-5MS (60 m) column, *split* 1:30, MSD (EI, 70 eV). Compound identification appears in **Table 1.**

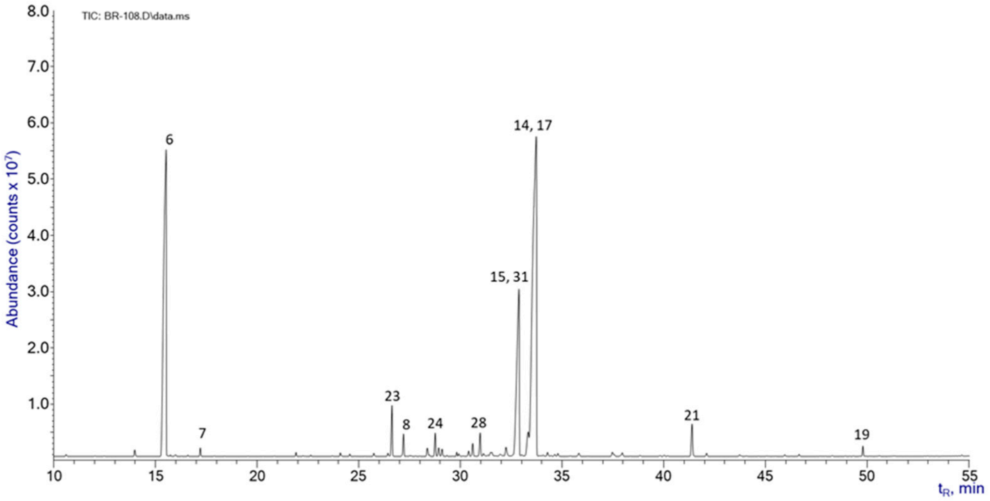

Figure S2. Chromatographic profile (GC/MS, full scan) of *Lippia alba* carvone chemotype, essential oil. DB-WAX (60 m) column, *split* 1:30, MSD (EI, 70 eV). Compound identification appears in **Table 1.**
